# Assessing the completeness of periodontal disease documentation in the EHR: a first step in measuring the quality of care

**DOI:** 10.1186/s12903-021-01633-w

**Published:** 2021-05-29

**Authors:** Joanna Mullins, Alfa Yansane, Shwetha V. Kumar, Suhasini Bangar, Ana Neumann, Todd R. Johnson, Gregory W. Olson, Krishna Kumar Kookal, Emily Sedlock, Aram Kim, Elizabeth Mertz, Ryan Brandon, Kristen Simmons, Joel M. White, Elsbeth Kalenderian, Muhammad F. Walji

**Affiliations:** 1 Willamette Dental Group, Portland, OR USA; 2 San Francisco – School of Dentistry, University of California, San Francisco, CA USA; 3 School of Dentistry, University of Texas Health Science Center At Houston, 7500 Cambridge, SOD 4184, Houston, TX 77054 USA; 4 Harvard School of Dental Medicine, Boston, MA USA; 5 School of Dentistry, University of Pretoria, Pretoria, South Africa

**Keywords:** Quality, Dental quality measures (DQMs), Value, EHR, Periodontal disease, Periodontal risk assessment

## Abstract

**Background:**

Our objective was to measure the proportion of patients for which comprehensive periodontal charting, periodontal disease risk factors (diabetes status, tobacco use, and oral home care compliance), and periodontal diagnoses were documented in the electronic health record (EHR). We developed an EHR-based quality measure to assess how well four dental institutions documented periodontal disease-related information. An automated database script was developed and implemented in the EHR at each institution. The measure was validated by comparing the findings from the measure with a manual review of charts.

**Results:**

The overall measure scores varied significantly across the four institutions (institution 1 = 20.47%, institution 2 = 0.97%, institution 3 = 22.27% institution 4 = 99.49%, *p*-value < 0.0001). The largest gaps in documentation were related to periodontal diagnoses and capturing oral homecare compliance. A random sample of 1224 charts were manually reviewed and showed excellent validity when compared with the data generated from the EHR-based measure (Sensitivity, Specificity, PPV, and NPV > 80%).

**Conclusion:**

Our results demonstrate the feasibility of developing automated data extraction scripts using structured data from EHRs, and successfully implementing these to identify and measure the periodontal documentation completeness within and across different dental institutions.

## Introduction

Modern-day healthcare places an increased emphasis on quality improvement to achieve better patient outcomes [1, 2]. Quality measures act as observable tools to evaluate the performance of healthcare processes against established standards of care, both at the program and practice level [3]. Alternative payment methods, e.g., Pay for Performance and “Value Based Care”, were in part developed to incentivize providers to achieve improved patient outcomes at lower costs. Regardless of the payment model in vogue, the importance of healthcare quality and the need for accurate and valid quality measures that can quantify healthcare performance are evident [4]

The Dental Quality Alliance (DQA) [5] has played a vital role in the development and promotion of dental quality measures (DQMs) [5–7]. Although the current methods of DQMs rely heavily on claims-based measures/administrative data, there has been a move towards harnessing Electronic Health Record (EHR) data to identify and report quality measures in dentistry [8, 9]. The passing of the Health Information Technology for Economic and Clinical Health (HITECH) Act aimed to encourage adopting and promoting of "meaningful use" of EHRs and has paved the way to developing EHR-based quality measures [10], including dentistry [11]. The inherently rich content of EHR data, which consists of critical patient-level information, makes it an excellent resource to measure healthcare performance. Numerous studies have reported high validity of electronic quality measures for assessing dental care [12–14].

Periodontal disease is a chronic inflammation of soft tissues and alveolar bone surrounding the teeth; when left untreated, the progressive loss of attachment may increase in tooth mobility and premature tooth loss [15]. Based on the 2009–2010 National Health and Nutrition Examination Survey (NHANES) cycle, 47% of the total US adult population suffered from periodontitis, while 64% of adults age 65 and older had either moderate or severe periodontitis [16]. Host-microbial interactions are key factors in the pathogenesis of periodontitis, while certain risk factors including smoking, poor oral hygiene, and diabetes are likely to increase host susceptibility [17, 18]. Clinical periodontal status parameters, including scores of supragingival plaque, bleeding, suppuration and probing depth as well as tissue appearance can be useful indicators of periodontal disease presence and progression [19]. Identifying an individual's risk factors could further enhance evaluation and monitoring of the susceptibility for disease progression, thereby optimizing treatment strategies [20, 21]. Based on this premise, developing and using accurate, reliable, and standardized risk assessment tools are paramount [17, 22].

In 2015, the DQA reported as part of its environmental scan, four periodontal health measures in use at the practice level [23], which represent the accepted standard of periodontal care among oral health care providers [24]. The DQA itself has one periodontal measure under development, which assesses the utilization and quality of periodontal care [23, 25]. While there are no universally accepted periodontal disease risk assessment (PDRA) tools in dentistry, there has been a movement to develop assessment tools that evaluate the risk for developing periodontal disease based on pre-specified conditions [22, 26, 27]. Currently, there is limited information available about the generalizability of these measures [28, 29].

As the first step towards our longer-term goal of determining the appropriateness of treatment and the outcomes of care, the aim of this study was to develop an EHR-based quality measure to determine how thoroughly different dental institutions document basic periodontal information. Our objective was to measure the proportion of patients for which a comprehensive periodontal charting, periodontal disease risk factors (diabetes status, tobacco use, and oral home care compliance) [22, 30–32], and periodontal diagnoses were documented in the EHR.

## Methods

Our study collected data from three academic institutions (Harvard School of Dental Medicine, UCSF School of Dentistry, and UTHealth School of Dentistry at Houston) and one large accountable dental care organization (Willamette Dental Group) that all used the same EHR system (Exan, Coquitlam, BC, Canada). Our research team, comprised of clinicians, informaticians, public health dentists, and statisticians, developed the following measure to assess and document periodontal disease risk and diagnosis.

### Study population

**Fig. 1:**
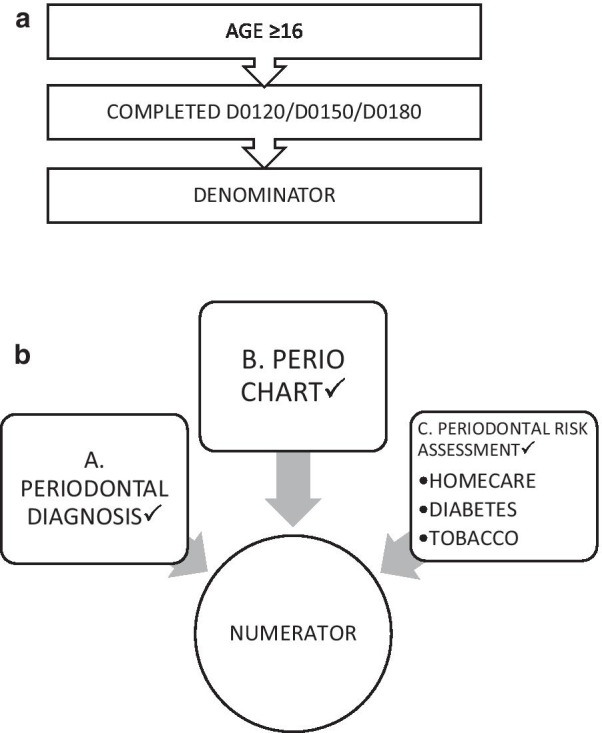
**a** Process map to determine the denominator. **b** Process map to determine the numerator

We designed our measures while considering existing practice guidelines regarding assessing periodontal risks. The *denominator* of the measure included patients 16 years of age or older and had at least one completed or in progress comprehensive/periodic/or periodontal exam (D0120/D0150/D0180) in the reporting year (Fig. 1a). Aged 16 and above are included in the periodontal measure because at this age the full complement of permanent teeth, except the 3^rd^ molars, are present and fully erupted. There is no agreed upon standard of when to begin periodontal charting. It is common clinical practice, to begin periodontal charting of pocket depths in patients at 16 years old, although depending on patients individual conditions, periodontal charting can begin sooner. For this measure, we set 16 years old as a common age that periodontal charting would be recorded.

The *numerator* included patients who had a completed periodontal probing charting, an assessment of all three periodontal disease risk factors (diabetes status, tobacco use, and oral home care compliance), and a periodontal diagnosis within six weeks of their comprehensive/periodic/or periodontal exam. The institutions that were part of this research study all use the SNODDS diagnostic terminology, which is a subset of SNODENT [33, 34] and the periodontal diagnostic terms that are included in it. SNODENT and SNODDS are in the process of updating the periodontal diagnostic terms to the most recent AAP classification system. As such this study includes the diagnostic terms that were part of the previous AAP classification (Fig. 1b and Appendix A) [35].

### Approach for testing and validating the automated query

Data from the 2015 calendar year was used for testing and validation purposes using the following steps:

- **Step 1** *Measure automation* (*automated query*) All institutions generated the sampling frame from their EHR using the same Structured Query Language (SQL) script, assuring all patients who were eligible would be included in both the denominator and the numerator.
- **Step 2*** Sample size estimation* We estimated the sample size using the proportion of patients who received periodontal charting, periodontal risk assessment, and periodontal diagnoses for each institution during the reporting period as derived from the automated query. We calculated the required sample size for a manual review with a precision of 5% around the expected effect size at the 95% confidence interval (CI) level.
- **Step 3** *Measure validation* We validated the automated query performance through a manual chart review, which served as the gold standard. In our earlier studies [13, 14, 36, 37], we effectively calibrated two trained reviewers (dentists or dental hygienists) at each institution, by calculating the interrater reliability using 50 manual chart audits. When both reviewers achieved > 80% agreement [38], we proceeded with single reviews to complete the remaining charts, calculated the sensitivity, specificity, positive predictive value, and negative predictive value of the automated query.
- **Step 4** *Measure score* EHR-measure proportions were calculated as a percentage of numerator/denominator for each institution.
- **Step 5** *Statistical analyses* By institution, descriptive analysis was employed for all measure scores. The frequencies and percent for the total number of patients who received tobacco, homecare (defined as the presence of heavy plaque), and diabetes screenings, had comprehensive periodontal charting and received periodontal diagnoses were calculated. Line graphs were generated to show institutional variations of the measure scores over time, and bar charts were generated to show the institutional distributions. In order to determine whether there were statistically significant differences between the measures scores calculated by manual review and those calculated by the query results, an independent sample hypothesis test was performed. All tests were conducted at the standard significance level of 0.05 (α = 0.05) and all analysis used Stata Statistical software release 14 for StataCorp LP. After the validation process, the measures were run again for three additional years (2016, 2017, and 2018).

## Results

The validity of the measure score was established using standard diagnostics (sensitivity, specificity, positive predicted value (PPV), and negative predictive value (NPV)). 205 charts were manually reviewed at Institution 1, 323 charts at institution 2, 312 charts at institution 3, and 384 charts at institution 4. The score derived from the manual chart reviews at each institution was compared with the score calculated from the automated query. Overall, the diagnostic tests showed a high sensitivity, specificity, PPV, and NPV across all institutions (Table 1). Institution 2 had the lowest PPV among the institutions, which we attributed to the low counts of patients in the denominator.

**Table 1:** Description of measure validity using manual reviewed charts as gold standard

	Institution 1	Institution 2	Institution 3	Institution 4
Kappa	0.8960	–	0.8960	1.000
Sensitivity	100%	100%	94.2%	100%
Specificity	95.8%	99.7%	97.2%	100%
PPV	83.7%	50%	94.1%	100%
NPV	100%	100%	97.2%	100%

Table 2 summarizes the patient sample and the results of the automated query across the four institutions. The mean age of the population for the caries risk e-measures was 42.7 years (SD = 16.6). For the overall measure score in 2018, Institution 1 had an "Overall Measure" score of 20.47%, indicating that only 1 in 5 charts contained all the necessary information; comprehensive periodontal probing chart, periodontal risk factors, and a periodontal diagnosis. To help identify gaps, Table 2 also shows the component measures comprising the full score. At Institution 1 for the year 2018, 42.71% of the 6452 patients in the denominator had a periodontal diagnosis documented, 62.95% had a comprehensive periodontal chart, and 40.07% had all three of the periodontal risk factors assessed. Of the periodontal risk factors assessed for Institution 1 in 2018, homecare was less frequently documented (45.29%), compared to diabetes (67.37%) and tobacco status (68.92%).

**Table 2:** Overall and component measure scores across the 4 institutions (n = number of patients meeting the denominator criteria in the respective reporting year)

	Overall measure (%)	A. Perio diagnosis (%)	B. Perio chart (%)	C. Perio risk (%)	C.1 homecare (%)	C.2 diabetes (%)	C.3 tobacco (%)
*2015*							
Institution 1 (n = 6825)	16.44	43.22	54.90	26.81	33.44	62.68	62.36
Institution 2 (n = 3590)	0.64	3.26	32.67	3.59	4.48	86.24	78.61
Institution 3 (11,296)	29.57	48.57	66.47	49.96	55.21	69.87	69.65
Institution 4 (142,690)	99.22	99.90	99.40	99.82	99.86	99.88	99.86
*2016*							
Institution 1 (n = 7379)	22.46	46.54	59.52	36.56	43.56	66.57	66.36
Institution 2 (n = 3762)	1.97	11.99	34.18	4.31	5.53	84.34	76.56
Institution 3 (n = 12,279)	26.58	43.85	65.65	51.20	56.16	69.62	69.39
Institution 4 (n = 151,824)	99.07	99.90	99.25	99.81	99.83	99.85	99.86
*2017*							
Institution 1 (n = 6853)	23.96	47.23	62.95	40.07	47.05	69.71	70.26
Institution 2 (n = 3938)	0.89	10.46	36.52	4.06	5.13	82.58	75.24
Institution 3 (12,989)	24.92	42.01	66.90	46.24	51.67	68.33	68.07
Institution 4 (166,139)	99.34	99.90	99.53	99.80	99.81	99.83	99.83
*2018*							
Institution 1 (n = 6452)	20.47	43.71	60.63	37.21	45.29	67.37	68.92
Institution 2 (n = 4411)	0.97	8.41	36	3.36	4.87	83.38	74.65
Institution 3 (n = 13,146)	22.27	37.86	69.57	46.18	52.69	67.62	67.12
Institution 4 (n = 177,441)	99.49	99.91	99.66	99.83	99.84	99.84	99.85

**Fig. 2:**
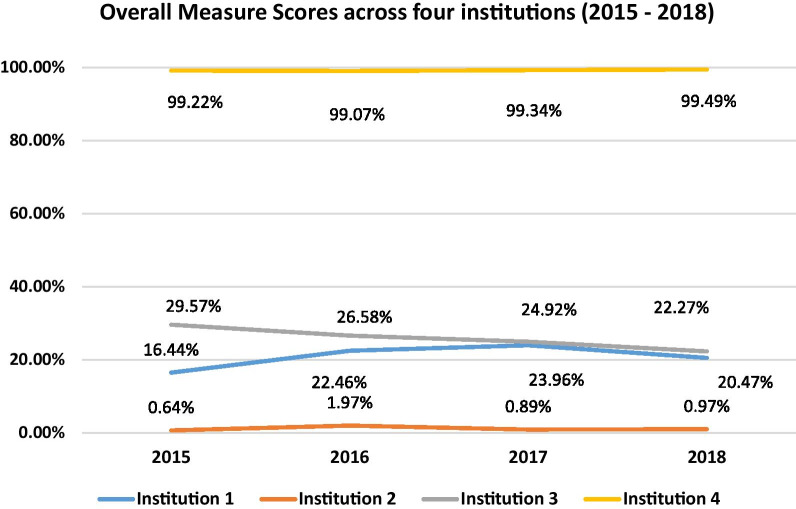
Comparison of overall measure scores across the four institutions for years 2015 to 2018

As shown in Fig. 2, the performance on the overall and component measures varied across the four institutions. Using a chi-squared test for homogeneity of proportions, there were significant variations in the measure score between institutions across all years. Institution 4 significantly outperformed the other three institutions, while Institution 2 consistently scored the lowest over the study period. (2015: χ^2^ = 1.2e05, *p*-value < 0.0001; Institution 3: χ^2^ = 1.2e05, *p*-value < 0.0001; Institution 1: χ^2^ = 1.4e05, *p*-value < 0.0001; Institution 4: χ^2^ = 1.6e0, *p*-value < 0.0001).

The chi-squared test for homogeneity of proportions, found significant variations in the overall measure score over time within the same institution. For Institution 1, the chi-squared test showed that the overall measure score in year 2017 was significantly higher than in other years, and there was a 4.03% decrease over the study period (Institution 1: χ^2^ = 132.6, *p*-value < 0.0001). For Institution 2 the chi-squared test shows the measure scores in 2016 (1.97%) were significantly higher than the other years for Institution 2 (Institution 2: χ^2^ = 34.7, *p*-value < 0.001). Institution 2 experienced a 0.3% increase over the 4-year study period. For Institution 3, the chi-squared test shows the measures scores in 2015 were significantly higher than those in other years (Institution 3: χ^2^ = 178., *p*-value < 0.0001). Lastly, the chi-squared test showed that Institution 4 measure scores in 2018 outperformed all other years (Institution 4: χ^2^ = 221.3, *p*-value < 0.0001), the scores remained relatively constant over time.

## Discussion

Our results demonstrate the feasibility of developing automated data extraction scripts using structured data from EHRs, and successfully implementing these to identify and measure the periodontal documentation completeness within and across different dental institutions. Even though all four institutions included in the study used the same EHR and the same standardized diagnostic terminology (SNODDS) [34, 39], we saw a large variation in how they performed on the measure. For instance, Institution 4, scored highly on all components, including a routinely captured periodontal diagnosis, comprehensive periodontal charts, and assessment of all three periodontal risk factors. Institution 2 rarely captured a periodontal diagnosis and homecare and therefore had the lowest overall measure score. We anticipate that these measures can assist institutions in identifying specific areas for more in-depth inquiry and improvement. For instance, when critical components of periodontal health status and care are captured in the EHR, we can then use this data, such as a diagnosis and risk factors, to measure and infer the appropriateness of periodontal treatment and outcomes of that care [40].

To date, the dental profession as a whole has not yet adopted a universal model of periodontal disease risk assessment, possibly due to challenges related to varying workflows, practice types, general agreement, adoption of EHRs, different types of EHRs, and continued challenges with interoperability [20, 41]. In our study, the research team identified and applied three factors known to be key to assessing periodontal disease risk: (1) diabetes status, (2) tobacco consumption, and (3) homecare status as measured by the presence of heavy plaque. We observed that diabetes status and tobacco use were generally well documented as the medical history forms are routinely updated. However, finding documentation of visible heavy plaque was more challenging. Some institutions incorporated a specific and structured question (e.g., "Does the patient have visible heavy plaque?") as part of their risk assessment form. While in other cases we extracted this information from a plaque score recorded as part of the periodontal exam. Our process and findings suggest the need for a standardized approach for assessing periodontal risk. A simple, universal risk assessment tool will benefit the profession [22], and further help to direct appropriate care [20, 42]. This can translate into creating a culture of quality improvement and accountability rather than a focus on treatment and payment [43]. Risk assessment measures also pave the way for the development of clinical decision support (CDS) tools [44], and thus allowing for the provision of more timely information to clinicians and patients [45].

According to the 1990–2010 Global Burden of Disease Study, periodontal disease ranks sixth in worldwide prevalence of Oral conditions in 2010 [46] and 11th as a preventable global disease. Periodontal disease causes a significant economic burden, with an estimated 54 billion USD/year of lost productivity and 3.5 million years lived with disability [47]. Periodontal disease has a higher prevalence among the ageing population due to longer life spans and higher retention of natural dentition in this age group. With the pressure of an aging dentate population [48], the rising understanding of the complexity of the disease [49], and its relationship with chronic diseases [50], there has been an increasing focus on understanding and managing the underlying risk factors [51]. Periodontal risk assessment strategies help evaluate and quantify risk [52], thus providing internal benchmarks to assess periodontal care and evaluate disease progress or lack thereof in patients [22, 28, 52, 53]. The work conducted here provides an approach for better documenting the periodontal status of patients which is the first step before determining appropriate treatment pathways and for measuring the outcomes of care [20, 41, 54]. Moreover, complete and accurate documentation in the EHR are the foundation for measurement in a value based care system, which dentistry undoubtedly will need to embrace [55].

## Limitations

Some of the data used to measure documentation completeness are self-reported (e.g., diabetes status and tobacco use). Although, self-reported questionnaires have shown to have high sensitivity, they are potentially inaccurate due to their subjective nature [56]. Meaningful integration and coordination between medical and dental records may greatly reduce these challenges [57]. Our measure will also be challenging to implement in dental institutions that currently lack a robust informatics infrastructure including a common EHR and diagnostic terminology. Large group dental practices, community dental clinics and academic dental clinics with the requisite infrastructure are likely to benefit by implementing such a measure. Other dental practices who have not yet adopted EHRs and a standardized diagnostic terminology may still be able to estimate periodontal disease documentation quality by conducting manual reviews on a randomly selected sample of patient charts. We also recognize that institutions may differ in their guidelines for the need and frequency for comprehensive periodontal charting; and the measure may need to be adjusted to conform to these local practices.

## Conclusion

The study results highlight variation between dental institutions in capturing essential data to measure and evaluate the completeness of periodontal disease documentation. This work supports the use of EHRs, standardized dental diagnoses, and the potential for dental quality measures to assess appropriate periodontal disease evaluation before treatment in an effort to optimize outcomes of care.

## Data Availability

Our legally binding data use agreements between the institutions do not allow public sharing of these data that are derived from electronic heath records. We are willing to consider reasonable requests for data access if the institutions agree and appropriate data use agreements are executed.

## Appendix A

**Table:**

**Periodontal diagnoses**
**Periodontal health**
Healthy periodontium
Healthy periodontium with attachment loss
**Necrotizing periodontal diagnosis**
Necrotizing ulcerative periodontitis
Necrotizing ulcerative gingivitis
**Developed/acquired def./cond**
Gingival soft tissue enlargement
**Gingival diagnosis—plaque induced**
Drug induced gingivitis-other (NOS)
Drug induced gingivitis—oral contraceptive associated
Gingivitis modified by malnutrition—ascorbic acid deficient
Leukemia associated gingivitis
Gingivitis associated with other blood dyscrasias
Diabetes mellitus associated gingivitis
Puberty associated gingivitis
Plaque induced gingival disease without local contributing factors
Plaque induced gingival disease with local contributing factors
Pregnancy associated gingivitis
Menstrual cycle associated gingivitis
Gingivitis modified malnutrition
**Aggressive periodontitis**
Prepubertal periodontitis
Post adolescent periodontitis
Juvenile periodontitis
Localized moderate aggressive periodontitis
Generalized moderate aggressive periodontitis
Rapidly progressive periodontitis
Localized slight aggressive periodontitis
Generalized slight aggressive periodontitis
Generalized severe aggressive periodontitis
Localized severe aggressive periodontitis
**Chronic periodontitis**
Generalized slight chronic periodontitis
Generalized severe chronic periodontitis
Localized moderate chronic periodontitis
Localized severe chronic periodontitis
Refractory periodontitis
Generalized moderate chronic periodontitis
Periodontosis
Localized slight chronic periodontitis
**Gingival diagnosis—non-plaque induced**
Gingivitis associated with recurrent oral herpes
Gingivitis associated with Candida
Gingivitis associated with primary herpetic gingivostomatitis
Gingivitis hereditary gingival fibromatosis
Gingivitis of genetic origin
Gingivitis associated with mucocutaneous disorder
Gingivitis associated with fungal origin
Gingivitis associated with *Treponema pallidum*
Dental restorative material—acrylic
Reactions attributable to foods and additives
Gingivitis associated with histoplasmosis
Reactions attributable to other
Reactions attributable to chewing gum additives
Gingivitis associated lichen planus
Gingivitis associated with lupus erythematosus
Gingivitis associated with Streptococcal species
Dental restorative material—other
Dental restorative material—nickel
Gingivitis associated with varicella zoster
Gingivitis associated with pemphigus vulgaris
Gingivitis with linear erythema of fungal origin
Gingival disease modified by Gingivitis Neisseria gonorrhea
Gingivitis with linear erythema multiforme
Dental restorative material—mercury
Gingivitis associated with pemphigoid
Gingivitis associated with viral origin
Reactions attributable to mouth rinses and mouthwashes
